# The impact of the LactApp m-Health tool on breastfeeding self-efficacy: a mixed methods study among Filipino mothers

**DOI:** 10.4069/whn.2025.06.02

**Published:** 2025-06-30

**Authors:** Rudena A. Madayag, Matthew Andrei E. Basilio, Dondon D. Pamintuan, Maria Luisa D. Galang, Jennifer Mae P. Malonzo-Rocero, Rolando L. Lopez Jr, Jerry Ligawen, Isabelito A. Nabong, Karen D. Sembrano, Milagros C. Si, Anita B. Viray

**Affiliations:** 1 College of Nursing and Graduate School, Angeles University Foundation, Angeles City, Philippines; 2 College of Nursing, Angeles University Foundation, Angeles City, Philippines

**Keywords:** Breast feeding, Lactation, Maternal health, Mobile applications, Self efficacy

## Abstract

**Purpose:**

Breastfeeding self-efficacy is essential for successful breastfeeding, and mobile health applications offer a promising approach for increasing maternal confidence. This study investigated the impact of LactApp (LactApp Women’s Health) on breastfeeding self-efficacy among Filipino mothers and explored their experiences and perceptions of the app as a support tool.

**Methods:**

A sequential explanatory mixed-methods design was utilized. The quantitative phase employed a single-group pre- and post-test design with 280 Filipino mothers who used LactApp for 3 months, measuring changes in breastfeeding self-efficacy. Because the data were not normally distributed, the Wilcoxon signed-rank test was conducted. The qualitative phase involved semi-structured interviews with 30 mothers, which were analyzed using thematic analysis. Integration of quantitative and qualitative findings was achieved through a joint display.

**Results:**

Quantitative analysis demonstrated a significant improvement in breastfeeding self-efficacy, with median scores increasing from 49 to 61 (Z=–3.20, *p*<.001) and reduced score variability. The largest improvements occurred among mothers aged 21 to 30 years and multiparous women. Qualitative findings corroborated these results, with mothers describing increased confidence, improved breastfeeding techniques, and solutions to challenges such as low milk supply. However, some participants, particularly those with limited educational backgrounds, experienced difficulties, indicating ongoing challenges related to digital access and app usability.

**Conclusion:**

LactApp improved breastfeeding self-efficacy among Filipino mothers, especially those with prior breastfeeding experience. The culturally relevant content and user-friendly design supported maternal confidence. To expand reach and impact, future interventions should address barriers to app use, especially for digitally underserved groups, and explore integration with traditional breastfeeding support systems.

## Introduction

Breastfeeding self-efficacy is a critical factor influencing breastfeeding success, affecting both initiation and duration. A mother’s confidence in her ability to breastfeed directly impacts breastfeeding outcomes; higher self-efficacy is associated with longer breastfeeding durations and improved maternal and infant health outcomes [1,2]. Self-efficacy, defined as an individual’s belief in their capacity to influence life events, is positively linked to breastfeeding practices and duration [3]. Research has shown that mothers with higher breastfeeding self-efficacy demonstrate greater confidence in breastfeeding techniques and decision-making [1]. Additionally, there is a positive correlation between the number of prenatal checkups and breastfeeding self-efficacy, suggesting that prenatal assessments can identify mothers at risk for early weaning challenges [4].

Li et al. [5] found that factors such as maternal education, feeding support, and delivery methods significantly affect breastfeeding self-efficacy. Recent studies indicate that targeted nursing interventions—including structured prenatal education, peer counseling, and digital health support—can substantially enhance maternal self-efficacy [6,7]. For instance, interventions that deliver personalized guidance, timely feedback, and continuous support empower mothers to overcome common breastfeeding difficulties and adopt effective feeding practices [8]. These approaches provide essential knowledge and skills, while also promoting confidence and resilience [9]. Evidence from various cultural contexts demonstrates that mothers who receive comprehensive support through both traditional counseling and real-time guidance exhibit improved breastfeeding initiation, longer durations, and higher exclusive breastfeeding rates [7,10]. This move toward multifaceted intervention strategies highlights the potential for comprehensive support systems to advance positive maternal and infant health outcomes [11].

Multiple studies underscore the role of breastfeeding self-efficacy in building maternal confidence. Greater breastfeeding confidence cultivates a positive attitude toward breastfeeding, thereby further enhancing self-efficacy [12]. Titaley et al. [13] observed that first-time mothers with extended maternity leave and previous breastfeeding experience had higher self-efficacy, facilitating sustained breastfeeding. Wong et al. [14] showed that interventions designed to increase self-efficacy led to higher exclusive breastfeeding rates at 4 and 8 weeks postpartum. Escribano et al. [15] noted that self-efficacy shapes mothers’ inclination to breastfeed, emphasizing the importance of self-control in feeding behaviors.

Nonetheless, research gaps remain. Few studies have investigated the long-term effects of breastfeeding self-efficacy beyond the immediate postpartum period. Additional cross-cultural validation of the Breastfeeding Self-Efficacy Scale-Short Form (BSES-SF) is needed, particularly in rural and underserved populations and diverse socioeconomic settings.

Although the BSES-SF is a well-validated measure, there is limited research examining the role of m-Health tools such as LactApp (LactApp Women’s Health, Barcelona, Spain) in enhancing self-efficacy, especially among Filipino mothers. This study addresses this gap by evaluating breastfeeding self-efficacy among Filipino postpartum mothers and examining the influence of prenatal care, socioeconomic status, and cultural factors on breastfeeding confidence and outcomes.

LactApp, a mobile application providing evidence-based breastfeeding information and personalized support, represents a promising approach to addressing gaps in breastfeeding support. During the coronavirus disese 2019 pandemic, the use of LactApp increased, highlighting its potential to provide critical guidance when in-person support was limited [16]. However, there remains little empirical evidence regarding its impact on breastfeeding self-efficacy among Filipino mothers [17].

This study aimed to answer the following research questions: to what extent does the use of LactApp improve breastfeeding self-efficacy among Filipino mothers? Addressing this research gap will inform the effectiveness of m-Health interventions in maternal and child health and support efforts to increase exclusive breastfeeding rates in the Philippines.

## Methods

**Ethics statement:** This study received approval from the Ethics Review Committee of Angeles University Foundation (2024-CON-Faculty-004) and is registered with ClinicalTrials.gov (Identifier: NCT06862661). All participants provided informed consent prior to participation. In accordance with ethical guidelines for vulnerable populations, assent was obtained in addition to written consent from a parent or legal guardian for mothers aged 16 to 17 years, ensuring that these participants were fully informed about the study’s procedures, risks, and their right to withdraw at any time.

### Study design

This research employed a sequential explanatory mixed-methods design, consisting of a quantitative phase using a single-group intervention design, followed by a qualitative phase with in-depth interviews and focus group discussions (FGDs). The quantitative phase was reported in accordance with the TREND (Transparent Reporting of Evaluations with Nonrandomized Designs) statement (https://www.equator-network.org/reporting-guidelines/improving-the-reporting-quality-of-nonrandomized-evaluations-of-behavioral-and-public-health-interventions-the-trend-statement/), while the qualitative phase followed the COREQ guidelines (https://www.equator-network.org/reporting-guidelines/coreq/).

### Quantitative phase

#### Setting and sample

**Figure 1.**
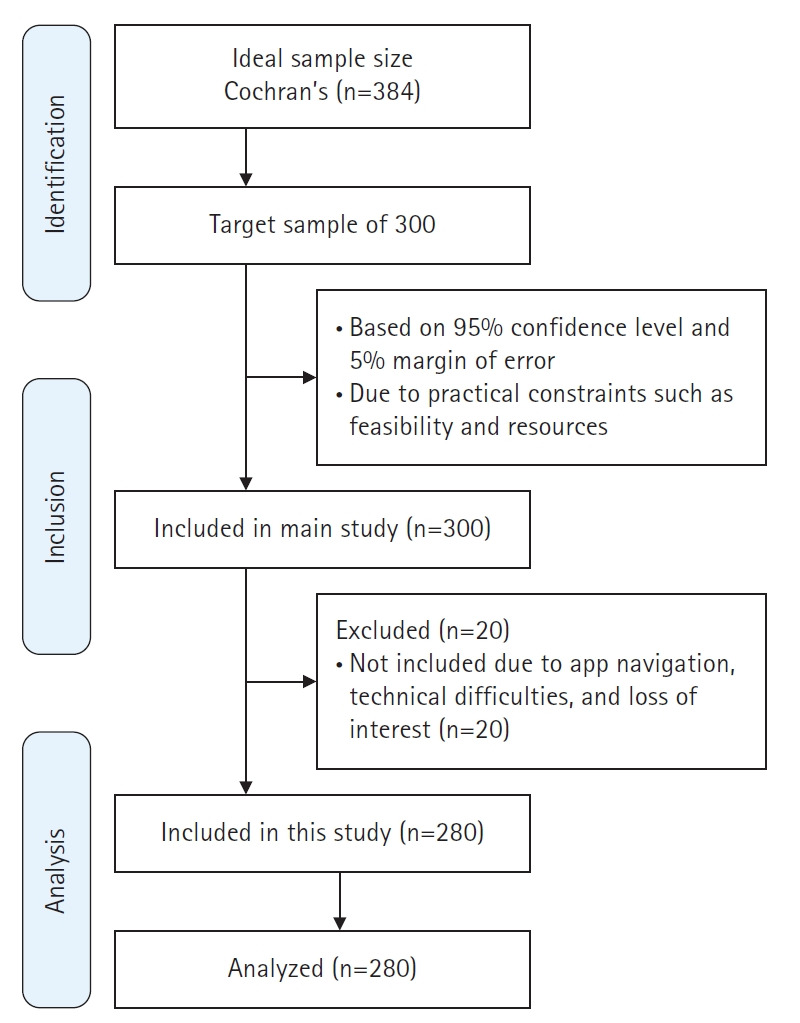
Flow diagram of participant recruitment.

The study was conducted in selected barangays in Angeles City, Pampanga, Philippines, chosen for their diverse populations and established community health programs, which facilitated participant recruitment. The target population included Filipino mothers aged 16 to 45 years who were pregnant or had children under the age of one year, as this group is most directly affected by breastfeeding challenges and maternal health concerns. Inclusion criteria required participants to be seeking breastfeeding support, open to using the LactApp tool, and willing to provide informed consent. Mothers who did not intend to breastfeed, had significant health issues preventing tool use, or were not fluent in the language of the app were excluded. A stratified random sampling method was used to ensure representation across key demographic factors such as age, socioeconomic status, and breastfeeding experience. The sample size was calculated using Cochran’s formula for categorical data, with a 95% confidence level and a 5% margin of error. A 95% confidence level was selected as standard in health research to ensure the true population parameter is within the calculated range 95% of the time, while a 5% margin of error was chosen to balance precision and recruitment feasibility. According to Cochran’s formula, a minimum of 384 participants was required. Ideally, to account for an anticipated attrition rate of approximately 20%, the recruitment target should have exceeded 384. However, due to practical constraints related to feasibility and available resources, only 300 mothers were targeted. Following dropouts resulting from app navigation issues, technical difficulties, or loss of interest, the final sample comprised 280 completed surveys (Figure 1).

A post-hoc power analysis was conducted using data from the Wilcoxon signed-rank test (Table 1), which compared pre-test and post-test breastfeeding self-efficacy scores. Based on the observed effect size (approximate Cohen’s d=0.39) and an alpha level of 0.05 (two-tailed), the analysis indicated that the study achieved a power of approximately 95%. This result confirms that, despite attrition, the final sample size was adequate to detect the observed improvements in breastfeeding self-efficacy.

#### Study variables and measures

Breastfeeding self-efficacy was measured using the BSES-SF [18], administered in English. Permission to use the BSES-SF was obtained from the original developers prior to data collection. The survey served as a baseline measure of mothers’ confidence in their breastfeeding ability. The BSES-SF uses a 5-point Likert scale, where participants rate their confidence from 1 (“strongly disagree,” lowest confidence) to 5 (“strongly agree,” highest confidence). Each statement reflects different aspects of breastfeeding tasks, such as positioning the baby or managing challenges, and respondents select the option that best matches their confidence in performing the task. Total scores range from 14 to 70. The internal consistency of the BSES-SF was excellent, with a Cronbach’s alpha coefficient of 0.910, indicating strong reliability. Furthermore, the validity of the BSES-SF is well established through confirmatory factor analyses and its demonstrated predictive value for breastfeeding initiation and duration across diverse cultural settings [19]. This robust evidence supports the instrument’s reliability and validity for assessing breastfeeding self-efficacy in this study.

Additionally, a structured questionnaire was used to collect sociodemographic data, including participants’ age, marital status, education, household income, employment, parity, birth experience, and current infant feeding practice. These variables were collected to facilitate subgroup comparisons and to contextualize changes in breastfeeding self-efficacy across participant groups.

#### Intervention: LactApp tool

The intervention in this study involved introducing the LactApp tool to participating mothers as a means to enhance breastfeeding self-efficacy. LactApp is a mobile application designed to provide personalized guidance and support to breastfeeding mothers through educational content, video tutorials, reminders, and expert advice, with the goal of improving the breastfeeding experience [20].

During clinical rotations at community health centers, the researchers—who also served as clinical instructors for students—introduced the study to potential participants. This familiar and trusted environment helped establish rapport and facilitated participant engagement from the outset. Researchers ensured that each mother had access to a smartphone or device capable of downloading the LactApp and provided step-by-step instructions on how to install the app and set up user profiles. Mothers were oriented to key app features, including:

● Breastfeeding tips: Daily tips and advice tailored to mothers’ breastfeeding journey

● Tracking tools: A tool to track breastfeeding frequency, duration, and baby’s weight

● Expert support: Access to a community forum for peer support, as well as direct consultations with lactation experts

Researchers emphasized the importance of regular app usage as part of the breastfeeding routine, highlighting LactApp’s ability to answer questions, provide reassurance, and deliver real-time feedback. The intervention period was standardized at 3 months, during which participants were encouraged to use LactApp consistently and were supported by weekly follow-ups. These check-ins monitored engagement, provided additional support when needed, and addressed any technical or usage issues.

#### Procedures

Data collection was conducted from September 5 to December 5, 2024. Eligible mothers were approached during routine visits to health centers and screened for inclusion. After obtaining informed consent, participants completed the pre-test survey (BSES-SF and sociodemographic questionnaire) in person.

Participants were then given access to the LactApp tool for a 3-month intervention period.

During student clinical rotations, researchers introduced the study to eligible mothers at community health centers and presented the LactApp tool as part of a structured breastfeeding support strategy. Mothers received step-by-step guidance on installing and using the app, including instructions on personalized advice, feeding logs, and community forums. Weekly in-person or mobile check-ins were conducted to maintain participant engagement and provide technical assistance as needed.

At the end of the 3-month period, participants completed the post-test survey, which readministered the BSES-SF to assess changes in breastfeeding self-efficacy.

#### Data analysis

Pre-test and post-test survey data were analyzed to assess changes in breastfeeding self-efficacy scores. Although regression analysis was initially considered to explore predictors of change, normality was first evaluated using the Shapiro-Wilk test. Results indicated significant deviations from normality for all survey items in both the pre-test and post-test datasets (*p*<.001; test statistics, 0.599–0.900), confirming that breastfeeding self-efficacy scores were not normally distributed. Accordingly, parametric methods, including regression analysis, were deemed inappropriate.

The Wilcoxon signed-rank test was therefore used to assess paired differences between pre-test and post-test scores. To interpret the magnitude of change across subgroups (e.g., age, parity, household income, educational attainment), the research team applied a classification framework based on Wilcoxon test statistics, including *p*-values, Z-scores, and effect size (r). Within this framework: improvements were classified as highly improved when the *p*-value was ≤.001, the Z-value was less than –2.00, and the effect size exceeded 0.50; improvements were deemed moderate when the *p*-value was ≤.05, the Z-value ranged between –1.96 and –2.00, and effect size was between 0.30 and 0.49; changes were categorized as low if the *p*-value exceeded .05 or effect size was below 0.30; and as no improvement when the *p*-value was non-significant with minimal or no change in Z-scores. This framework guided subgroup result labeling in tables, such as “low” improvement for the high-income group or mothers with no formal education.

A post-hoc power analysis based on the Wilcoxon test (Table 1) showed an approximate Cohen’s d of 0.39, indicating a moderate effect size. With an alpha of 0.05 (two-tailed), the study achieved a statistical power of about 95%, confirming that the final sample size of 280 was sufficient to detect meaningful changes.

Complete case analysis was employed, including only participants with both pre-test and post-test data. Participant dropout was documented, but no imputation or sensitivity analyses were performed. All statistical analyses were conducted using SPSS version 29, and results were interpreted at a significance threshold of *p*<.05.

For subgroup analysis, educational attainment categories were standardized according to the Philippine education system: “post-secondary” referred to diploma or technical-vocational credentials, while “tertiary” included bachelor’s and postgraduate degrees.

### Qualitative phase

#### Sample

Following 3 months of LactApp use and completion of the post-test survey, purposive sampling was employed based on demographic variations identified in the quantitative findings. Eligible mothers were personally invited to participate through face-to-face invitations during scheduled visits to barangay health centers or through home visits conducted by the research team and community health nurses.

During the invitation process, the study’s purpose, procedures, and the voluntary nature of participation in interviews or FGDs were clearly explained. A total of 30 mothers consented to participate, with 10 taking part in in-person interviews and 20 participating in FGDs (organized into four groups of four to six participants each). Qualitative data analysis was conducted concurrently, and no further participants were invited once data saturation was reached.

#### Procedures

Each interview and FGD session lasted 20 to 30 minutes and followed a semi-structured guide reviewed by maternal and child health experts. The chief investigator (RAM) conducted the interviews, supported by five co-researchers (DDP, MLDG, JMPMR, JL, and RLL), and assisted by nursing students during their clinical placements. The students facilitated logistics and participant comfort but were not directly involved in the interviews or data analysis to minimize bias. All sessions were conducted in English or Filipino, based on participant preference, and were held at either barangay health centers or the mothers’ homes, according to convenience. Interviews were audio-recorded, transcribed, and translated into English by the research team during the final phase. All facilitators maintained a neutral, non-judgmental stance to preserve data integrity. No repeat interviews were conducted.

#### Qualitative data analysis

Thematic analysis was performed according to Braun and Clarke’s six-phase framework [21]. A hybrid approach was used: deductive coding based on the interview guide and quantitative findings was combined with inductive identification of emerging themes. While the interview guide provided initial categories, the analysis extended beyond descriptive grouping to uncover deeper patterns and emotional insights.

Transcripts were independently coded by multiple researchers (RAM, RLL, JL, IAN, and KDS), reviewed by MLDG and JMPMR, and verified by experts MCS and ABV. Any discrepancies were discussed and resolved collaboratively during 13 research meetings. Despite time constraints, the interviews produced rich, meaningful, and reflective data.

Member checking was conducted a week after the interviews, during which participants reviewed selected themes in follow-up meetings. All 30 participants confirmed the accuracy of their transcripts, enhancing the credibility of the findings.

### Integration phase

The integration phase aimed to build upon the quantitative results by using qualitative insights to provide context, explanation, and depth [22]. A joint display was used as the primary integration strategy [23]. This visual matrix enabled a side-by-side comparison of statistical trends from the quantitative phase with thematic findings from the qualitative phase. Through this approach, patterns of integration were identified, including alignment (where qualitative data confirmed statistical improvements), explanation (where participant narratives clarified subgroup variations), expansion (where qualitative themes introduced new insights not captured by the survey), and discordance (where inconsistencies arose between the two data strands).

Integration occurred at the interpretation and reporting stage, with a focus on determining whether the qualitative findings supported, explained, or diverged from the quantitative results.

## Results

### Quantitative results

Table 2 provides a comprehensive overview of participants’ demographic characteristics and their breastfeeding self-efficacy scores before and after using the LactApp m-Health tool. Of the 280 mothers, most were aged 21–30 years (57.2%), with a high proportion married (57.5%) and multiparous (60.4%). Educational attainment varied: 50% had completed high school and 25.4% held a college degree or higher. The majority of participants reported an average household income (58.9%), and most delivered by normal spontaneous delivery (65.0%). Mixed feeding was the most common feeding practice (38.9%), followed by partial breastfeeding (27.9%), exclusive breastfeeding (20.4%), and formula feeding (12.9%).

Statistical analysis with the Wilcoxon signed-rank test revealed a significant overall increase in breastfeeding self-efficacy following the 3-month intervention. As shown in Table 1, the median self-efficacy score increased from 49 at pre-test to 61 at post-test, with a test statistic of Z=–3.20 (*p*<.001). The mean score rose from 45.97 (SD, 14.56) to 59.43 (SD, 7.77). Additionally, the interquartile range narrowed from 19.75 to 9, and the score range shifted from 56 points (14–70) to 39 points (31–70), indicating increased consistency and reduced variability in participant responses.

Improvements were seen consistently across most demographic subgroups. Mothers aged 21–25 and 26–30 years showed highly improved median scores (from 50 and 52 to 65, respectively), with large effect sizes (r=.76 and r=.70). Marked improvements were also observed among multiparous mothers (r=.66), those with tertiary education (r=.81), and participants from middle-income households (r=.72), all statistically significant at *p*<.001.

However, the analysis also identified variation in intervention effectiveness. Mothers with no formal education (n=5) and those with separated marital status (n=2) showed low or no improvement, with *p*-values >.05 and smaller effect sizes. Exclusive breastfeeding participants demonstrated moderate gains (r=.29, *p*=.031), while formula-feeding mothers showed substantial improvement (r=.85, *p*<.001).

In summary, these results confirm that the LactApp intervention significantly enhanced breastfeeding self-efficacy across most demographic groups. The observed positive shifts in central tendency and reduced variability further suggest that the tool contributed not only to greater confidence but also to a more uniform sense of self-assurance among participating mothers.

### Qualitative results

Table 3 presents four major themes and 11 subthemes derived from interviews with mothers regarding their experiences with LactApp. These themes illustrate how the app supported their breastfeeding journey in practical, emotional, and behavioral ways.

**Theme 1 focused on how the app empowered mothers through knowledge. Participants described an increased understanding of breastfeeding techniques, greater confidence, and a stronger sense of control over their decisions. One mother shared,** “*I didn’t realize there were so many ways to improve my milk supply until I used the app*” (P9, age 25 years). Another added, “*Having reliable information at my fingertips made me feel more capable*” (P11, age 30 years), emphasizing how real-time access to credible information strengthened their self-efficacy.

**Theme 2 captured the emotional support mothers received from the app. The ability to access immediate help relieved anxiety and made participants feel less alone.** “*The app made me feel calmer because I could get answers right away*,” noted one participant (P14, age 26 years). Others described the app as a comforting companion: “*It’s like having a friend who understands and wants to help you*” (P17, age 28 years). A sense of community also emerged. As one mother explained, “*Knowing other moms were using it made me feel I wasn’t alone*” (P18, age 27 years).

**Theme 3 focused on the development of healthy breastfeeding habits. The app played a role in helping mothers persevere through breastfeeding challenges, address common problems, and gradually prepare for weaning.** “*The app helped me keep breastfeeding, even when it was hard*,” said one mother (P19, age 25 years), while another appreciated the assistance with specific issues: “*The app helped me when I had sore nipples*” (P21, age 26 years). The guidance provided a sense of structure and encouragement, especially for mothers transitioning out of breastfeeding: “*The app explained how to stop breastfeeding step by step*” (P23, age 27 years).

**Theme 4 reflected opportunities for app improvement based on user suggestions. Participants recommended adding real-time expert support for more personalized guidance.** “*It would be nice to chat with an expert in the app for extra advice*,” suggested one mother (P25, age 29 years). Others highlighted the importance of inclusivity through multilingual options: “*It would be much better if the app had different languages, like Filipino*” (P27, age 31 years). Some also expressed a need for advice tailored to special cases, such as breastfeeding twins or infants with health conditions: “*It would be really useful if the app had more advice for moms like me…*” (P30, age 34 years).

Although not directly tied to the study aims, many mothers emphasized LactApp’s intuitive design and ease of use as key facilitators of their positive experience. Comments included, “*The app is simple to use. I didn’t have trouble finding what I needed, like the section on latching problems*” (P1, age 29 years), and “*The layout is clear, and I could quickly find what I was looking for, even on my first try*” (P3, age 26 years). Others noted the quick help, “*Whenever I had a question, I got answers right away—it saved me a lot of time*” (P5, age 32 years) and “*It gave me answers to my questions fast, especially when I needed help at night*” (P8, age 24 years). Participants also appreciated the personalized features: “*I liked that the app gave tips based on my baby’s age and feeding habits*” (P6, age 31 years), and “*It gave me suggestions based on my specific situation, like how to pump milk while at work, it made me feel supported*” (P2, age 27 years). These reflections highlight the significance of user-friendly features in improving digital health engagement.

### Integration phase results

Table 4 presents the integration of quantitative findings and qualitative themes, providing a comprehensive understanding of how the LactApp m-Health tool influenced breastfeeding self-efficacy among Filipino mothers. This joint display approach revealed patterns of alignment, complementarity, expansion, and discordance between statistical outcomes and experiential narratives.

The study demonstrated a statistically significant improvement in breastfeeding self-efficacy following the use of the LactApp m-Health tool. Quantitative results showed that median BSES-SF scores increased from 49 at pre-test to 61 at post-test, with the Wilcoxon signed-rank test confirming statistical significance (Z=–3.20, *p*<.001). There was also a reduction in the interquartile range (from 19.75 to 9), reflecting greater consistency in participant responses.

Alignment was observed among mothers aged 21 to 30 years and those who were multiparous. These groups experienced significant improvements in BSES-SF scores, which were mirrored in qualitative narratives describing increased breastfeeding knowledge, improved decision-making, and a stronger sense of competence and confidence while using the app. These responses align with Theme 1, particularly Subthemes 1 and 2, emphasizing the app’s role in empowering mothers through knowledge and reinforcing existing skills.

Complementarity emerged in participants with no formal education and among separated mothers. Quantitative data in these subgroups showed minimal to no improvement. Qualitative accounts, however, provided important context: mothers with no formal education described challenges in navigating the app due to limited literacy and digital skills (Theme 4: Subtheme 2), while separated mothers reported time constraints and lack of household support as barriers to consistent app use (Theme 2: Subtheme 3). These insights clarified the observed statistical trends and highlighted the importance of supportive design and external support mechanisms.

Expansion was seen in domains not captured by the BSES-SF. Many mothers described feeling emotionally supported, less anxious, and more connected with other users through the app. These emotional and psychosocial benefits—represented in Theme 2: Subthemes 1, 2, and 3—were not directly measured by the quantitative tool but contributed an essential dimension to understanding user experience. In particular, the sense of peer support and shared stories fostered a community atmosphere that encouraged continued breastfeeding.

Discordance appeared in groups where quantitative outcomes did not reflect positive qualitative feedback. For example, exclusive breastfeeding mothers exhibited only moderate statistical gains but reported high levels of satisfaction and motivation to continue breastfeeding, suggesting a qualitative–quantitative mismatch (Theme 3: Subtheme 1). Similarly, high-income participants showed only modest score improvements but expressed a preference for face-to-face consultations, indicating that digital solutions may not fully meet their expectations (Theme 4: Subtheme 2).

Overall, this integration demonstrates that, while LactApp significantly enhanced breastfeeding self-efficacy on average, the tool’s impact varied across subgroups. The integrated findings suggest directions for future improvement, including simplifying app design, addressing emotional needs, and incorporating personalized features tailored to users’ lived experiences and contextual realities.

## Discussion

This study demonstrated the value of integrating quantitative and qualitative methods to assess the impact of a culturally contextualized m-Health tool, LactApp, on breastfeeding self-efficacy among Filipino mothers. The mixed-methods approach allowed for both the measurement of statistically significant changes in self-efficacy and the development of a rich understanding of users’ experiences, perceptions, and contextual challenges.

The alignment between improved self-efficacy and participants’ narratives—particularly among younger and multiparous mothers—confirms the relevance of LactApp for key maternal demographics. These findings are consistent with previous studies demonstrating that digital health tools are most effective when they complement prior knowledge or coincide with periods of high motivation in the maternal journey [24,25]. This alignment reinforces the role of mobile interventions as timely reinforcements for skill-building and confidence.

Importantly, this study uncovered nuanced outcomes not fully captured by quantitative data alone. Emotional reassurance, reduced anxiety, and a sense of community emerged as prominent qualitative themes—dimensions often overlooked in standard self-efficacy assessments. These psychosocial benefits align with earlier research on m-Health in postpartum care, which highlights the dual role of digital tools in delivering both information and emotional support [26,27]. Furthermore, similar findings have underscored how supportive interactions with healthcare professionals, peer groups, and family members can significantly shape mothers’ self-perception and confidence throughout the breastfeeding journey [28].

In cases where numerical improvements were less pronounced, particularly among mothers with no formal education or separated marital status, qualitative data provided critical explanatory context. These groups frequently struggled with app navigation and had difficulty comprehending digital content, which limited their ability to fully benefit from the intervention. Structural barriers, such as unstable home environments and lack of social support, further reduced their engagement. These findings underscore the critical influence of technological familiarity and contextual vulnerabilities on the effectiveness of m-Health tools. The complementary insights from qualitative analysis reinforce the need to tailor digital interventions for diverse user capacities. This highlights ongoing calls for simplification, clearer instructions, and the integration of offline or community-linked support mechanisms, particularly in low- and middle-income settings [29,30].

Discordant patterns also emerged. For example, high-income mothers and those exclusively breastfeeding demonstrated only moderate statistical gains, yet they reported high satisfaction with the app’s practical support. While some users preferred more personalized, face-to-face care, others valued LactApp’s accessibility and convenience. These perspectives suggest that digital health interventions should incorporate flexibility and adaptability to address a range of user expectations and preferences [24].

Participants also provided constructive feedback for app improvement, such as the inclusion of real-time expert consultations, multilingual content, and tailored advice for specific breastfeeding situations. These suggestions echo inclusive design principles recommended by Rivera et al. [31], reinforcing the importance of involving users in the co-design process to promote accessibility, engagement, and equity.

Despite these promising outcomes, this study has several limitations. Reliance on self-reported data introduces potential bias, and the relatively short duration of the study limits the ability to assess long-term behavioral changes. The exclusion of 20 participants in the per-protocol analysis may have narrowed the breadth of findings. Expanding to an intention-to-treat analysis and incorporating longer-term follow-up would provide a more comprehensive picture of the app’s sustained impact.

Looking ahead, m-Health developers and implementers should emphasize inclusive and user-centered design. This includes simplifying app interfaces, providing multilingual and culturally relevant content, and supplementing digital tools with in-person or community-based support. Evidence from other low- and middle-income countries suggests that m-Health can play a valuable role in strengthening maternal and child health services, even in the face of infrastructural and educational barriers [32]. Longitudinal research will be essential to determine whether initial gains in self-efficacy translate into sustained breastfeeding practices and improved maternal–infant health outcomes.

In conclusion, use of the LactApp over a 3-month period was associated with increased breastfeeding self-efficacy among Filipino mothers, achieved by combining practical advice with emotional support. The greatest impact was observed among younger, more experienced, and better-educated mothers. To achieve broader equity and effectiveness, future m-Health strategies should prioritize reaching marginalized populations through adaptive, accessible, and culturally responsive innovations.

## Figures and Tables

**Table 1.** Pre- and post-tests for breastfeeding self-efficacy according to general characteristics (N=280)

Variable	Categories	n (%)	Median		Z-value	*p*-value	Effect size (r)
			Pre-test	Post-test			
Age (year)	16–20	27 (9.6)	49	61	–3.51	<.001	–.68
	21–25	80 (28.6)	50	65	–6.72	<.001	–.76
	26–30	80 (28.6)	52	65	–5.53	<.001	–.70
	31–35	57 (20.4)	55	66	–4.51	<.001	–.60
	36–40	36 (12.9)	57	68	–3.81	<.001	–.64
Marital status	Married	161 (57.5)	51	66	–8.44	<.001	–.66
	Single	117 (41.8)	49	64	–7.19	<.001	–.67
	Separated	2 (0.7)	45	50	–1.34	.180	–.47
Highest educational attainment	No formal education	5 (1.8)	47	53	–1.83	.068	–.82
	Primary school	20 (7.1)	48	54	–0.83	.408	–.19
	Secondary school	140 (50.0)	50	65	–7.41	<.001	–.63
	Post-secondary (diploma, associate’s degree)	44 (15.7)	52	66	–4.73	<.001	–.71
	Tertiary education (bachelor’s, postgraduate)	71 (25.4)	55	68	–6.83	<.001	–.81
Household income	Low	105 (37.5)	50	65	–5.81	<.001	–.57
	Middle	165 (58.9)	51	66	–9.21	<.001	–.72
	High	10 (3.6)	53	64	–2.45	.014	.78
Parity	Nulliparous	111 (39.6)	51	66	–8.06	<.001	–.77
	Multiparous	169 (60.4)	50	65	–7.47	<.001	–.66
Birth experience	Normal Spontaneous	182 (65.0)	51	67	–8.68	<.001	–.64
	Caesarean	77 (27.5)	52	64	–5.34	<.001	–.61
	Others	21 (7.5)	49	58	–3.93	<.001	–.86
Infant feeding status	Partial breastfeeding	78 (27.9)	50	63	–4.59	<.001	–.52
	Exclusive breastfeeding	57 (20.4)	52	62	–2.16	.031	–.29
	Mixed feeding	109 (38.9)	51	64	–8.23	<.001	–.79
	Formula feeding	36 (12.9)	53	66	–5.09	<.001	–.85

**Table 2.** Changes in breastfeeding self-efficacy of mothers after using LactApp (N=280)

Measure	Pre-test	Post-test
Median	49	61
Interquartile range	19.75	9
Range	56 (14–70)	39 (31–70)
Mean	45.97	59.43
Standard deviation	14.56	7.77
Z-value	–	–3.20
*p*-value	–	<.001

**Table 3.** Mothers’ experiences and perspectives on the use of LactApp m-Health tool

Theme	Subtheme	Significant quotes
1. Empowerment through knowledge	1. Increased knowledge	*“I didn’t realize there were so many ways to improve my milk supply until I used the app.” (P9, age 25 years)*
		*“The app taught me things I didn’t even hear from the doctor.” (P4, age 30 years)*
	2. Confidence in breastfeeding	*“When I felt like I wasn’t doing enough, the app reminded me that I was on the right track.” (P7, age 28 years)*
		*“When I felt overwhelmed, it gave me hope to keep trying.” (P10, age 33 years)*
	3. Sense of empowerment	*“Having reliable information at my fingertips made me feel more capable.” (P11, age 30 years)*
		*“It made me feel like I could handle breastfeeding.” (P13, age 27 years)*
2. Emotional support	1. Feeling less worried	*“The app made me feel calmer because I could get answers right away.” (P14, age 26 years)*
		*“I felt less anxious knowing I had reliable help at my fingertips.” (P15, age 34 years)*
	2. Feeling cared for	*“It felt like someone was there to guide me, which made me happy.” (P16, age 29 years)*
		*“It’s like having a friend who understands and wants to help you.” (P17, age 28 years)*
	3. Support systems	*“Knowing other moms were using it made me feel I wasn’t alone.” (P18, age 27 years)*
3. Developing healthy breastfeeding habits	1. Continuing breastfeeding	*“The app helped me keep breastfeeding, even when it was hard.” (P19, age 25 years)*
		*“The reminders and encouragement in the app gave me the push I needed to stick with breastfeeding…” (P20, age 30 years)*
	2. Solving breastfeeding problems	*“The app helped me when I had sore nipples....” (P21, age 26 years)*
		*“I found helpful advice on how to improve my diet...” (P22, age 33 years)*
	3. Gradual weaning	*“The app explained how to stop breastfeeding step by step…” (P23, age 27 years)*
		*“The app gave clear guidance on how to stop breastfeeding slowly...” (P24, age 28 years)*
4. Opportunities for app enhancement	1. Adding expert advice	*“It would be nice to chat with an expert in the app for extra advice.” (P25, age 29 years)*
		*“A live chat feature with lactation experts would be extremely beneficial...” (P26, age 30 years)*
	2. Catering to diverse needs	*“It would be much better if the app had different languages …” (P27, age 31 years)*
		*“It would be really useful if the app had more advice for moms like me…” (P30, age 34 years)*

LactApp m-Health tool: LactApp Women’s Health, Barcelona, Spain.

**Table 4.** Integrated findings

Quantitative finding	Qualitative finding	Integration
Significant improvement in BSES-SF scores among mothers aged 21–30 (r=0.758, *p*<.001)	Participants reported increased confidence and knowledge after using the app (Theme 1: Subthemes 1 and 2**)**	Alignment: Quantitative gains among mothers aged 21–30 were reflected in qualitative reports of increased knowledge and confidence in breastfeeding techniques, showing alignment with narrative data.
Multiparous mothers had substantial improvements (r=0.657, *p*<.001)	Experienced mothers felt that the app enhanced existing knowledge and reaffirmed practices (Theme 1: Subtheme 1)	Alignment: Improvement in effect size among multiparous mothers was reinforced by reports of enhanced learning and validation of prior breastfeeding knowledge in experienced mothers in the narrative data.
Minimal improvement among mothers with no formal education (r=–0.817, *p*=0.068)	Participants expressed challenges with app navigation and understanding due to limited literacy (Theme 4: Subtheme 2)	Complementarity: The low statistical effect was contextualized by qualitative accounts describing challenges with app navigation and understanding due to limited educational background, with suggestions for simpler, language-accessible features.
Separated mothers showed no significant improvement (n=2, *p*=.180)	Participants in unstable households described limited time and support to engage with the app (Theme 2: Subtheme 3)	Complementarity: Narratives indicating limited time, unstable home environments, and lack of support systems clarified the quantitative finding on lack of improvement among separated mothers.
	Mothers described feeling less anxious and more emotionally supported during breastfeeding (Theme 2: Subthemes 1 and 2)	Expansion: Although not captured in BSES-SF scores, mothers consistently reported reduced anxiety and increased emotional reassurance, adding a new dimension to the app’s impact.
	Mothers valued peer stories and support features of LactApp (Theme 2: Subtheme 3)	Expansion: Qualitative insights revealed that peer support and shared experiences fostered a sense of belonging, a benefit not measured in the quantitative findings.
The exclusive breastfeeding group showed only moderate gains (r=0.286, *p*=.031)	Some exclusive feeders still reported high satisfaction and usefulness of the app (Theme 3: Subtheme 1)	Discordance: Despite modest statistical changes, some exclusive breastfeeding mothers expressed strong satisfaction and motivation to continue breastfeeding, reflecting a qualitative–quantitative mismatch.
The high-income group showed only moderate improvement (n=10, *p*=.014)	These mothers shared feedback that they preferred personalized in-person care (Theme 4: Subtheme 2)	Discordance: Limited quantitative gains were explained by high-income mothers’ preference for face-to-face consultations, suggesting that app-based tools may not fully meet their expectations.

LactApp m-Health tool: LactApp Women’s Health, Barcelona, Spain.
